# The Role of Tissue-Specific Ubiquitin Ligases, RNF183, RNF186, RNF182 and RNF152, in Disease and Biological Function

**DOI:** 10.3390/ijms21113921

**Published:** 2020-05-30

**Authors:** Takumi Okamoto, Kazunori Imaizumi, Masayuki Kaneko

**Affiliations:** Department of Biochemistry, Graduate School of Biomedical and Health Sciences, Hiroshima University, 1-2-3 Kasumi, Minami-ku, Hiroshima 734-8553, Japan; toka@hiroshima-u.ac.jp (T.O.); imaizumi@hiroshima-u.ac.jp (K.I.)

**Keywords:** RNF183, RNF186, RNF182, RNF152, ubiquitin ligase, RING finger, mTOR, NF-κB, endoplasmic reticulum stress, osmotic stress

## Abstract

Ubiquitylation plays multiple roles not only in proteasome-mediated protein degradation but also in various other cellular processes including DNA repair, signal transduction, and endocytosis. Ubiquitylation is mediated by ubiquitin ligases, which are predicted to be encoded by more than 600 genes in humans. RING finger (RNF) proteins form the majority of these ubiquitin ligases. It has also been predicted that there are 49 RNF proteins containing transmembrane regions in humans, several of which are specifically localized to membrane compartments in the secretory and endocytic pathways. Of these, *RNF183*, *RNF186*, *RNF182*, and *RNF152* are closely related genes with high homology. These genes share a unique common feature of exhibiting tissue-specific expression patterns, such as in the kidney, nervous system, and colon. The products of these genes are also reported to be involved in various diseases such as cancers, inflammatory bowel disease, Alzheimer’s disease, and chronic kidney disease, and in various biological functions such as apoptosis, endoplasmic reticulum stress, osmotic stress, nuclear factor-kappa B (NF-κB), mammalian target of rapamycin (mTOR), and Notch signaling. This review summarizes the current knowledge of these tissue-specific ubiquitin ligases, focusing on their physiological roles and significance in diseases.

## 1. Introduction

Ubiquitin (Ub) is a 76-amino-acid protein that is highly conserved among all eukaryotes. Ubiquitylation, which involves the conjugation of ubiquitin to the lysine residues of various cellular proteins, is one of the most prevalent post-translational modifications of proteins, and is usually catalyzed by a three-enzyme cascade consisting of Ub-activating enzymes (E1s), Ub-conjugating enzymes (E2s), and Ub ligases (E3s). In mammals, there are 10 or fewer E1 activating enzymes, dozens of E2 conjugating enzymes, and hundreds of E3 ligases; these enzymes regulate the ubiquitylation of numerous proteins [1]. In ubiquitylation, E1 initially activates ubiquitin by adenylating it at the C-terminal glycine residue in an adenosine triphosphate-dependent process; this activated ubiquitin is then captured by the catalytic cysteine of the E1, forming a thioester intermediate. Then, the thioester ubiquitin is transferred from the enzyme active site of E1 to the catalytic center cysteine residue of E2 via a trans-thioesterification reaction [2]. Finally, E3 mediates the transfer of ubiquitin from E2 to a substrate protein. Both the efficiency and the substrate specificity of the ubiquitylation reaction depend on E3 ligases. Depending on the mechanism by which ubiquitin is transferred from E2 to the substrate, E3 is classified into three broad families: Really Interesting New Gene (RING) finger domain-, Homologous to E6-associated protein C Terminus (HECT) domain-, or RING Between RING (RBR) domain-containing ubiquitin ligases. While RING E3 ligases, the major family among them, facilitate the direct transfer of ubiquitin from E2–ubiquitin intermediates to the substrate protein [3], HECT and RBR E3 ligases contain an active-site cysteine that forms a thioester with ubiquitin before transferring it to the substrate protein [4,5]. The selective pairing between E2 and the multiple cognate E3s confers the specificity necessary for the regulation by ubiquitylation of various biological pathways.

Ubiquitin is conjugated with a lysine residue of a substrate via its C-terminal carboxyl group and can also attach itself via the N-terminal methionine (M1) and seven lysine residues (K6, K11, K27, K29, K33, K48, and K63). As a result, a substrate protein is modified with a single monoubiquitin, multiple monoubiquitins, or polyubiquitin chain. Such ubiquitin attachments can be reversed in the process of deubiquitylation by deubiquitinase (DUB) [6]. Owing to the reversible nature of this modification, the ubiquitin pool of cells is divided into different fractions, including free monoubiquitins, covalently linked mono- and polyubiquitin–protein complexes, and unanchored polyubiquitin. These different linkage types and lengths affect substrate proteins in different biological and biochemical ways and play an essential role in regulating a considerable number of significant cellular functions (e.g., protein degradation, endocytosis of membrane proteins, transcriptional control, DNA repair, and cell cycle regulation) [7]. The principal and abundant forms are K48-linked and K63-linked polyubiquitin chains. K48-linked polyubiquitin functions as a signal of proteasomal degradation, whereas K63-linked polyubiquitin chains have non-degradative roles in cellular signaling, intracellular trafficking, the DNA damage response, and other contexts [8,9].

**Figure 1:**
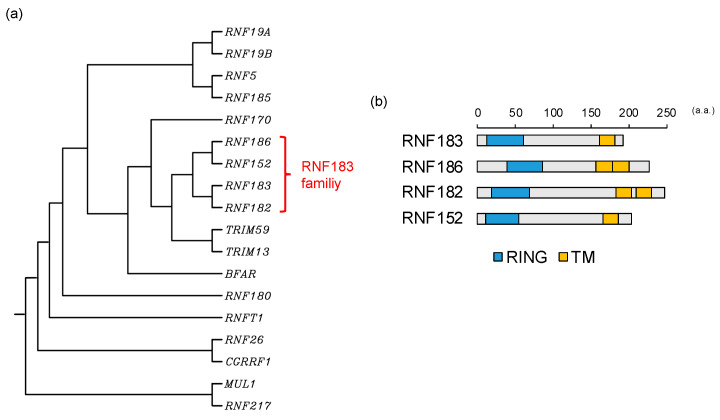
RNF183 family: (**a**) The phylogenetic tree for C3H2C3-RING E3s with transmembrane. Protein sequences for E3s were aligned with a multiple sequence alignment using the CLUSTALW (http://www.genome.jp/tools/clustalw); (**b**) The comparison of the domain structures of the RNF183 family. Information on the domain structure of RNF183 family protein was obtained from UniProt (https://www.uniprot.org) for RNF183 (Q96D59), RNF186 (Q9NXI6), RNF182 (Q8N6D2), and RNF152 (Q8N8N0). RING, C3H2C3-RING domain; TM, transmembrane domain; a.a., amino acids.

In this review, we focus on E3 ligases, *RNF183*, *RNF186*, *RNF182*, and *RNF152*, which are closely related genes encoding a RING-finger domain (C3HC4) at its N-terminus and one or two predicted transmembrane domains at its C-terminus with high homology (Figure 1) [10]. As common features, these E3s are expressed in specific tissues, such as the kidney, nervous system, and colon, and are localized in the lysosome (Table 1) [10]. Hereinafter, these ubiquitin ligases are referred to as the RNF183 family. In this review, we summarize our current understanding of the molecular mechanisms underlying the functions and regulation of these E3s in diseases.

## 2. RNF183

The E3 ubiquitin ligase RNF183 has been identified as a new biomarker of endometrial carcinoma (EC) via gene expression screening and protein level experiments on carcinoma samples. Furthermore, the differential expression of RNF183 in primary endometrial tumors has been shown to be correlated with its expression level in corresponding uterine fluid samples and it exhibits an analogous value in the initial stage of EC [22]. EC is the most common invasive tumor of the female genital tract, which is usually detected in its initial stages. However, 20% of patients are at an advanced stage at the time of detection. Because molecular markers for the diagnosis of EC have yet to be validated, new methods for the medical prognostication and classification of EC are needed to combat this deadly disease. RNF183 could be helpful as a precise molecular tool to diagnose EC and reduce unnecessary biopsies.

Another study has indicated that RNF183 interacts with fetal and adult testis-expressed 1 (FATE1) in tumors and negatively regulates the apoptosis effector Bcl-2-interacting killer (BIK), leading to increased viability of tumor cells [15]. FATE1 is one of the cancer/testis antigens whose expression is biased to the testes but is also activated in cancer [49,50]. Depletion of FATE1 reduces the viability of cancer cells. Large-scale proteomic studies have revealed that BIK is a FATE1-interacting partner. At the same time, RNF183 has also been revealed to be a FATE1-interacting partner. BIK associates with both FATE1 and RNF183, and both RNF183 depletion and the mutant RNF183, which exhibits the loss of enzyme activity, increase BIK protein accumulation. Thus, FATE1 and RNF183 collaborate to suppress BIK protein levels and escape from BIK-related apoptotic signaling [15]. However, in Ewing sarcoma cells, no appreciable levels of BIK protein are detectable even in the presence of the proteasome inhibitor MG132, and FATE1 depletion does not induce BIK accumulation.

There is another context-selective mechanism in Ewing sarcoma. FATE1 is most robustly induced by the Ewing sarcoma breakpoint region 1-Friend Leukemia Integration 1 (EWSR1-FLI1) chimeric transcription factor caused by a pathognomonic chromosomal translocation of Ewing sarcoma and interacts with Bcl-2/adenovirus E1B 19 kDa protein-interacting protein 3-like (BNIP3L). Then, BNIP3L is degraded in the presence of RNF183 [16]. Because BNIP3L is a tumor suppressor [51], its depletion increases tumorigenesis in vivo [16].

Moreover, *RNF183* has been identified as a gene conferring resistance to trametinib. Trametinib is one of the anticancer drugs inhibiting MEK1/2 [52]. RNF183 expression is increased after trametinib treatment, which in turn activates the NF-κB pathway. Then, the activated NF-κB increases the expression of the pro-inflammatory cytokine interleukin-8 (IL-8), which is a downstream target of NF-κB [20]. IL-8 signaling increases the proliferation and survival of cancer cells and potentiates their migration [53]. Thus, RNF183 confers resistance to trametinib on colorectal cancer (CRC) cells and promotes their proliferation and metastasis [20].

Under physiological conditions, RNF183 is not expressed in the large intestine, but is specifically expressed in the kidney [10]. The abnormal expression of RNF183 is thought to be involved in several diseases, not only tumorigenesis but also inflammatory conditions such as inflammatory bowel disease (IBD), including Crohn’s disease (CD) and ulcerative colitis (UC), which is a chronic, idiopathic, inflammatory, gastrointestinal disease, the molecular mechanism underlying the development and pathophysiology of which have not been fully elucidated. [15,16,17,20,21,22]. However, FATE1 is not expressed in the intestine. Therefore, there is a FATE1-independent inflammatory mechanism involving RNF183 in the large intestine. In fact, some studies have shown that RNF183 is upregulated in colon samples of the intestinal tissues of IBD patients [17] and the colons of mice with colitis treated with trinitrobenzene sulfonic acid (TNBS) or dextran sulfate sodium (DSS) [17,18].

It has been reported that RNF183 is largely involved in executing apoptosis in response to prolonged ER stress. It is considered that the mechanism of apoptosis involving RNF183 features the ubiquitylation and degradation of B-cell lymphoma extra-large (Bcl-xL), which functions as an inhibitor of apoptosis by preventing cytochrome c release [11]. Bcl-xL is usually localized to the mitochondria [54], whereas RNF183 is predominantly localized to the ER, Golgi, and lysosome [12]. Some Bcl-xL may be targeted to the ER [55], where it is in the vicinity of RNF183. Then, since their cytosolic domains can interact with each other, they interact directly and RNF183 ubiquitylates Bcl-xL [11]. The detailed mechanism behind this involves inositol requiring 1α (IRE1α) being activated by prolonged ER stress and readily decreasing microRNA-7 (miR-7) and microRNA-96 (miR-96), presumably by the digestion of miR precursors through the IRE1-dependent decay of mRNA [56,57]. Since miR-7 and miR-96 negatively regulate RNF183 by directly interacting with its 3′-UTR [17], their decrease eventually stabilizes the RNF183 mRNA and leads to increased protein levels. This increase in RNF183 in turn promotes its binding to Bcl-xL, polyubiquitylation, and subsequent degradation. The gradual decrease in Bcl-xL levels eventually triggers the intrinsic apoptotic pathway [11]. It has also been reported that increased RNF183 due to decreased miR-7 may contribute to the pathogenesis of IBD by recognizing NF-κB inhibitor α (IκBα), not Bcl-xL, as a substrate and degrading ubiquitylated IκBα [17]. Because IκBα is a suppressor of NF-κB, the reduction of IκBα by ubiquitylation and degradation induces NF-κB activation.

Recently, another mechanism of RNF183-related IBD pathogenesis has also been reported. Specifically, RNF183 recognizes DR5 as a substrate protein and K63-ubiquitylated DR5 is transported to lysosomes for degradation. In addition, RNF183 promotes TRAIL-induced caspase activation and apoptosis, providing new insights into the potential roles of RNF183 in DR5-mediated caspase activation in the pathogenesis of IBD [18]. RNF183-mediated ubiquitylation of substrates, Bcl-xL, IκBα, and DR5, and the negative regulation of RNF183 by miR-7 may be important novel epigenetic mechanisms in the pathogenesis of IBD.

In human and mouse tissues, RNF183 is specifically expressed in the kidney [10]. In particular, high Rnf183 expression in the renal medullary collecting duct has been reported from a tissue analysis using GFP-knock-in mice [13]. The kidney is the only tissue that is continuously under hypertonic conditions, and this hypertonicity gradually increases from the outer medulla down to the inner medulla. Nuclear factor of activated T cells 5 (NFAT5)/tonicity-responsive enhancer-binding protein is a transcription factor essential for the adaptation to hypertonic conditions, under which it stimulates the transcription of some genes [58]. The Rnf183 gene is also downstream of NFAT5 [14]. Indeed, the expression of Rnf183 in the renal medulla is dramatically decreased upon treatment with the loop diuretic furosemide, which can downregulate NFAT5 levels by inhibiting the Na-K-Cl cotransporter type 2 (NKCC2) and inducing hypotonicity in the medulla [13]. This is consistent with the decrease in NFAT5 protein and the mRNA expression of its target gene. Additionally, Rnf183 expression increases markedly in mouse inner-medullary collecting duct (mIMCD-3) cells treated with hypertonic NaCl. Rnf183, as well as several NFAT5 downstream genes, protects renal medullary cells from hypertonicity-induced apoptosis. mIMCD-3 cells transfected with siRNA targeting Rnf183 exhibit significant increases in cleaved caspase-3 protein levels. Therefore, Rnf183 expression is involved in the osmotic tolerance of mIMCD-3 cells [14].

In terms of its subcellular localization, RNF183 is predominantly localized to the endoplasmic reticulum (ER), Golgi apparatus and lysosome. Its stability depends on its interaction with Sec16A, which is involved in the formation of coat protein complex II (COPII) vesicles. However, Sec16A is not ubiquitylated by RNF183 [12]. Recently, it has been identified that the Na,K-ATPase β1 subunit, which forms a complex with Na,K-ATPase α1 subunit on the plasma membrane, is one of the substrates for RNF183. Na,K-ATPase contributes to the regulation of cell volume and solute absorption by the active transport of Na^+^ and K^+^ across the plasma membrane [59], and the expression of both α1 and β1 subunits is increased for adapting hypertonic condition in human renal cells [60]. RNF183 ubiquitylates only the β1 subunit, not the α1 subunit. Then, a complex with α1 and β1 subunits translocates from the plasma membrane to the lysosome, where it is degraded [19]. Therefore, RNF183 may play an important role in the kidney for the adaptation to hyperosmotic stress by regulating the level of Na,K-ATPase.

As described above, the kidney-specific ubiquitin ligase RNF183 protects cells from apoptosis induced by hypertonic stress in the kidney, whereas its aberrant expression such as in the colon induces inflammatory and tumorigenesis. Understanding the function of RNF183 could lead to new therapeutic strategies for patients with IBD and various types of cancer.

## 3. RNF186

A study of the distribution of RNF186 has indicated that its expression is highest in the lower gastrointestinal tract in both human and mouse tissues [10]. Initially, the RNF186 gene was identified as a locus associated with the risk of UC by several genome-wide association studies (GWAS) [27,28,29,30]. However, its function has only recently been clarified. Recently, deep resequencing of GWAS loci identified a causal variant in RNF186 that encodes an alanine-to-threonine substitution at position 64 [31]. This variant confers risk for the development of UC. The A64T variant is located in the RING-finger domain of RNF186. Since the RING-finger domain possesses E3 ubiquitin-protein ligase activity, there is the possibility that the A64T variant exhibits a reduction or complete loss of enzyme activity. A report indicates that RNF186 is thus a candidate for an association with the development of chronic inflammation in the intestine of UC. In addition, a recent study focusing on protein-truncating variants identified a novel genetic variant in RNF186, R179X [32]. This variant contributes to protection against UC. The R179X truncation is expressed at reduced levels and is diffusely localized, not in the ER, but preferentially in the plasma membrane [32]. Studying the specific effects of this variant, which has not yet been performed, should be useful for understanding the mechanism by which it protects against UC. In humans, RNF186 is also expressed in the kidney [10]. Interestingly, R179X truncation, which confers protection against UC, has also been identified as a factor associated with an increased risk of chronic kidney disease (CKD) [34].

Several RNF186 substrates have been identified, for example, Bcl2/adenovirus E1B 19-kDa interacting protein 1 (BNip1), occludin, and Sestrin-2 [23,24,25]. BNip1, a Bcl-2 family protein, co-localizes with and binds to RNF186 in the ER. RNF186 conjugates polyubiquitin through K29 and K63 linkage to BNip1 and the ubiquitylated BNip1 is transferred from the ER to the mitochondria [23]. Because BNip1 can induce a moderate level of apoptosis [61], RNF186 may regulate ER stress-induced apoptosis by the ubiquitylation of BNip1. The overexpression of RNF186 upregulates the critical regulators in the unfolded protein response (UPR), such as chaperone protein BiP/GRP78 and pro-apoptotic transcription factor CHOP/GADD153. Additionally, RNF186 triggers caspase-12 activation, which plays a role in inducing apoptosis in ER stress. Ca^2+^ is a secondary messenger of ER stress, and RNF186 actually promotes Ca^2+^ leakage from the ER [23]. Moreover, the expression of Rnf186 has been shown to be induced in the livers of mice with diabetes, obesity, and diet-induced obesity. In mouse primary hepatocytes, the overexpression of Rnf186 increases the protein levels of the ER stress markers, IRE1 and CHOP, as well as the level of eIF2α phosphorylation, and the treatment with tauroursodeoxycholic acid, the inhibitor of ER stress, decreases the expression of ER stress markers. It has been indicated that the overexpression of Rnf186 induces ER stress. These effects interfere with insulin action through the phosphorylation of insulin receptor substrate 1 (IRS1) by c-Jun N-terminal kinase (JNK). Furthermore, it has also been shown that the expression levels of proinflammatory cytokines, including TNF-α, IL-6, and MCP1, are increased by overexpressing Rnf186. Thus, Rnf186 may be a novel therapeutic target for the treatment of metabolic diseases associated with insulin resistance [26].

Occludin is one of the tight junction proteins, which control gastrointestinal tract permeability. In the colonic epithelia of mice with Rnf186 knockout, the expression of occludin protein is increased and its distribution is changed, resulting in increased permeability of small organic molecules. Rnf186 ubiquitylates occludin through K48-linked chains. Because K48-linked polyubiquitylation contributes to regulating protein turnover through proteasomal degradation, it is consistent with the increased expression of occludin in colonic epithelia of Rnf186 knockout mice. Disordered protein homeostasis in Rnf186 knockout mice correlates with enhanced ER stress in colonic epithelium and increased susceptibility to intestinal inflammation by DSS treatment. Therefore, it has been suggested that RNF186 is an E3 ubiquitin ligase controlling the homeostasis of occludin and ER stress in the colon [25].

Sestrin-2 is one of the intracellular amino acid sensors and functions as an inhibitor of mTORC1 signaling by interacting with GAP activity toward Rags 2 (GATOR2) [62]. This function occurs in a cytosolic leucine-dependent manner. When leucine is abundant in the cytosol, interference of the interaction between Sestrin-2 and GATOR2 occurs, allowing the activation of GATOR2. Because GATOR2 negatively regulates the GAP (GTPase-activating protein) activity of GATOR1, which inhibits mTORC1 activity, the decrease of Sestrin-2 indirectly activates mTOR1. Although Sestrin-2 has been shown to be transcriptionally controlled by several mechanisms, the regulation of the post-translational degradation of Sestrin-2 remains unclear. A recent study has shown that RNF186 and Sestrin-2 bind to each other via C-terminal motifs and RNF186 polyubiquitylated lysine residue at position 13 in Sestrin-2 with K48 linkage [24]. The ubiquitylated Sestrin-2 is degraded through the proteasome system. This is a new mechanism regulating mTORC1 activity through E3 ligases. In addition, Sestrin-2 also contains polyubiquitin chains with K63 linkages. Although this type has been shown to be involved in various processes, such as cellular trafficking, the role of the K63-linked ubiquitylation of Sestrin-2 remains unknown.

## 4. RNF182

RNF182 is the member of the RNF183 family on which the fewest reports have been published. It was initially identified as an upregulated gene in postmortem brains of patients with Alzheimer’s disease (AD) [35]. RNF182 is selectively expressed in the nervous system, such as the cortex, hippocampus, cerebellum, and spinal cord, but not in the heart, liver, kidney, and skeletal muscle. RNF182 is expressed in differentiated Ntera2 (NT2) neurons and was shown to be upregulated by oxygen and glucose deprivation. RNF182 overexpression can initiate the death of neuronal cells. In addition, yeast two-hybrid screening revealed that V-type proton ATPase 16 kDa proteolipid subunit (ATP6V0C) is a substrate of RNF182. RNF182 promotes the degradation of ATP6V0C via the proteasome pathway. Because ATP6V0C is a key component of gap junctions and neurotransmitter release channels, the upregulation of RNF182 in AD brains might induce ATP6V0C degradation and contribute to the pathophysiology of AD.

Next, RNF182 was identified as a gene associated with Rett syndrome. Patients with Rett syndrome often exhibit heterozygous mutations in the methyl-CpG-binding protein 2 (MECP2) gene encoding a transcriptional modulator [63]. Gene expression profiles obtained by microarray analysis revealed that RNF182 is upregulated in mutant MeCP2 fibroblasts obtained from patients with Rett syndrome [36]. Since, as described above [35], RNF182 possibly compromises brain function, it is speculated to be involved in postnatal neurodevelopmental abnormalities associated with Rett syndrome.

Although RNF182 appears to be specifically expressed in the nervous system, its expression and function in other tissues were reported. Rnf182 was also shown to be upregulated in a rat model of myocardial ischemia–reperfusion injury (MIRI) [37]. Its silencing by shRNA was shown to reduce myocardial infarct size and myocardial cell apoptosis in this rat model. Intriguingly, RNF182, as well as RNF152 [43,44] and RNF186 [24], is associated with the mTOR signaling pathway, since Rnf182 silencing and treatment with phosphoesterase, an inhibitor of the mTOR signaling pathway, reverse the effect of Rnf182 silencing in the MIRI rat. Thus, it is likely that Rnf182 silencing activates the mTOR signaling pathway, resulting in improved ventricular remodeling after MIRI. However, the target substrate of RNF182 was not identified in this study.

The expression of RNF182 has been observed in immune tissues such as lymph nodes and spleen and in immune cells such as macrophages and dendritic cells [38], although the levels of expression were not compared with that in nerve tissues. Lipopolysaccharide, poly(I:C) and CpG, which is involved in Toll-like receptor (TLR) signaling, induced RNF182 expression. Moreover, RNF182 silencing can specifically suppress TLR-induced activation of NF-κB and the NF-κB-mediated production of proinflammatory cytokines. RNF182 mediates K48-linked polyubiquitylation of the subunit p65 of NF-κB and its degradation via the proteasome pathway in macrophages. Therefore, RNF182 may be upregulated by immune responses and involved in the feedback of inflammatory responses.

## 5. RNF152

In terms of studies on RNF152, it was first reported as having decreased expression in breast and prostate cancer cell lines [45]. Next, RNF152 was cloned and characterized as a ubiquitin ligase [40]. It is localized in lysosomes and its overexpression can mediate self-polyubiquitylation through K48 linkage and induce apoptosis. However, its substrates and function initially remained unknown.

For the first time among members of the RNF183 family, RNF152 has been reported to be involved in mTOR signaling [43]. The ubiquitylation of RagA, which belongs to the Rag family of small GTPases and recruits mTORC1 to the lysosomal membrane [62], is markedly increased in response to amino acid starvation. RNF152 was identified among some lysosomal ubiquitin ligases and ubiquitylated RagA in a K63-linked manner. Increased amino acid levels facilitate the interaction of RNF152 with RagA and RNF152-mediated RagA ubiquitylation. RNF152 selectively ubiquitylates the inactive form of RagA (RagA-GDP/RagC-GTP), but not the active form (RagA-GTP/RagC-GDP), suggesting that the interaction between RNF152 and RagA is regulated by the nucleotide-bound status of RagA. Furthermore, the authors carefully demonstrated that RNF152 acts as a negative regulator of amino-acid-induced mTORC1 activation. RNF152-mediated RagA ubiquitylation promotes the binding between RagA and GATOR1, a GAP complex for Rag GTPases [62], resulting in the inactivation of RagA. USP17L2/DUB3 (ubiquitin carboxyl-terminal hydrolase 17), a deubiquitylating enzyme, was identified for RagA ubiquitylated by RNF152. Moreover, RNF152 knockout revealed that the deficiency of RNF152 causes the hyperactivation of mTORC1 and inhibits amino-acid-starvation-induced autophagy.

Four years later, the same group demonstrated that RNF152 is involved in the ubiquitylation of Rheb, another mTORC1 signal molecule, in growth factor-induced mTORC1 activation, not amino acids [62]. Rheb acts as a small GTPase like RagA and activates mTORC1 in its GTP-bound form. RNF152 induces Rheb monoubiquitylation that enhances binding to TSC2, which is a core subunit of the TSC complex and functions as a GAP [62], resulting in mTORC1 inactivation. On the other hand, USP4 was identified as a DUB for Rheb [64]. USP4 promotes the activation of Rheb by removing ubiquitin from it. USP4 activity is regulated by the EGF-Akt-mediated phosphorylation at S445. Therefore, RNF152 and USP4 can regulate Rheb activity negatively and positively, respectively, downstream of the EGF pathway. Since Rheb ubiquitylation negatively regulates mTORC1 activation, RNF152 can regulate cellular autophagy positively and cell proliferation negatively. Studies of USP4 knockout mice revealed that USP4 upregulates tumor growth in an mTORC1-dependent manner. In contrast, TCGA database indicates that RNF152 expression is downregulated in various types of cancer, including colon, lung, kidney, and liver cancers.

RNF152 in addition to RNF183 is also associated with CRC. 1,2-Dimethylhydrazine (DMH) is a potent carcinogen that acts as a DNA methylating agent. Microarray gene expression analysis showed that Rnf152 was upregulated in DMH-injected CRC model mice provided with high-calcium feed, compared with that in those provided with normal feed [46]. An additional relationship of RNF152 with CRC was reported in mTORC1 signaling [47]. RNF152 expression is significantly reduced in CRC tissues compared with that in normal tissues. The expression levels of RNF152 were reported to be correlated with prognosis in patients with CRC. Using cell lines and xenografts, it was demonstrated that RNF152 overexpression significantly decreased CRC cell growth in vitro and in vivo. RNF152 inhibits CRC cell proliferation by suppressing mTORC1, resulting in the induction of autophagy and apoptotic cell death. Although the ubiquitylation of RagA by RNF152 was not demonstrated in CRC cells, this finding is consistent with RNF152-mediated mTORC1 downregulation.

Rnf152 was first revealed to play a physiological role using zebrafish embryos [41]. The rnf152 transcript is now known to be expressed from the one-cell stage (maternally) to 48 h post-fertilization (hpf) (zygotically). Rnf152 is ubiquitously expressed in the brain at 24 hpf, whereas its expression is restricted to the eyes, midbrain–hindbrain boundary (MHB), and rhombomeres at 48 hpf. Since Rnf152 knockdown in zebrafish embryos leads to morphological defects in the eyes, MHB, and rhombomeres at 24 hpf, Rnf152 is required for appropriate development of the eyes and neural tube later than 18 hpf during embryogenesis. NeuroD is a marker for the inner and outer layers of the eyes at 48 hpf and plays a crucial role in regulating cell cycle exit and cell fate determination in mitotic cells [65,66]. NeuroD expression in rnf152-deficient embryos disappears in the marginal zone, outer nuclear layer (ONL), inner nuclear layer (INL), and ganglion cell layer (GCL) of the eyes at 27 hpf. Furthermore, the expression of deltaD and notch1a in rnf152 morphants is remarkably reduced in the ONL, INL, subpallium, tectum, and cerebellum [67]. Knockdown of rnf152 was found to cause decreases of the expression of her4 and ascl1a, Notch target genes [68], in specific regions at 24 hpf. Taking these findings together, Rnf152 may play essential roles in the development of the eyes, midbrain, and hindbrain, as well as the activation of Delta-Notch signaling. However, Deng et al. have already reported that Rnf152 knockout mice exhibited no difference in birth rates from the expected Mendelian ratios, suggesting that RNF152 is not required for embryonic development, at least in mouse [43]. Although these findings suggest that ubiquitin ligases other than Rnf152 can work in the neural development of mammals, it remains unknown whether Rnf182, which is also expressed in the nervous system, works compensatorily.

One study reported on the relationship between the RNF152–mTORC1 axis and neural development [42]. The cell proliferation rate of the floor plate in the ventral region of the neural tube remains low [69]. Forkhead-type transcription factor FoxA2 was demonstrated to be a negative regulator of mTORC1 signaling in the floor plate [70]. Furthermore, RNF152 was identified as a target gene for FoxA2 among mTORC1 signaling genes. The silencing of RNF152 causes aberrant mTOR activation and aberrant cell division in the floor plate. Therefore, RNF152 may function as a negative regulator for the mTORC1 signaling in the floor plate downstream of FoxA2.

## 6. mTORC1 in Cancer

**Figure 2:**
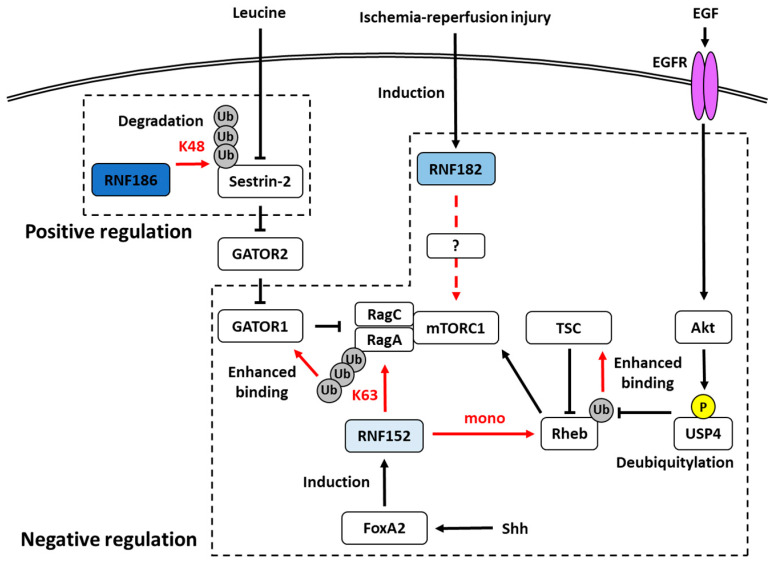
RNF186, RNF182, and RNF152 are involved in the regulation of the mTORC1 pathway. RNF186 regulates positively by degrading Sestrin-2 ubiquitylated with K48-linked chain, whereas RNF182 and RNF152 are native regulators. RNF152 involves two pathways: the K63-linked ubiquitylation of RagA and the mono ubiquitylation of Rheb. The ubiquitylated RagA and Rheb enhance the binding performance with GATOR1 and TSC, respectively. Phosphorylated USP4, which is identified as a DUB for Rheb, deubiquitylates the ubiquitylated Rheb and cancels the negative regulation by mono ubiquitylation. USP4 is phosphorylated by the EGF-Akt pathway. Shh-dependent FoxA2-transcriptional activity induces RNF152 expression. The expression of RNF182 is induced by ischemia-reperfusion injury; however, the mechanism of mTORC1 suppression by RNF182 remains unknown. The arrows indicate activation, and the T-bars indicate inhibition. The red arrows indicate the effects of ubiquitylation by RNF186 or RNF152. The red characters indicate the types of ubiquitylation by RNF186 or RNF152. The dotted arrow indicates that the detailed mechanism is unknown.

A common feature of members of the RNF183 family, with the exception of RNF183, is the ability to regulate mTORC1 signaling in the lysosomal membrane, albeit in different manners. RNF152 [43,44] and RNF182 [37] negatively regulate it, whereas RNF186 [24] positively regulates it (Figure 2). Since mTORC1 signaling is important for cell growth [62], these E3s may be involved in cancer progression. Indeed, members of the RNF183 family are known to be commonly associated with cancer (Table 1). As demonstrated by the findings for RNF152 [44,47], the expression levels of these E3s may affect cell proliferation by regulating mTORC1. Moreover, members of the RNF183 family, except RNF186, are frequently associated with CRC. The expression of RNF183 and RNF182 is upregulated in CRC [21,39], whereas that of RNF152 is downregulated [44,47]. This tendency is largely consistent with the findings for other cancers in TCGA database. Thus, the expression levels of these E3s may also be associated with the progression of other cancers besides CRC. Further investigations are needed to determine whether members of the RNF183 family are involved in other cancers, which are regulated by mTORC1. The mTORC1 signal is currently attracting attention as a target for drug discovery, and mTOR inhibitors such as everolimus are actually in use as molecular-targeted drugs for various cancers [62]. Therefore, if the mTORC1 signal of the RNF183 family and its involvement in cancer are clarified, they have potential as targets for new anticancer agents.

## 7. NF-κB and NFAT5 in Inflammation and Osmotic Stress

**Figure 3:**
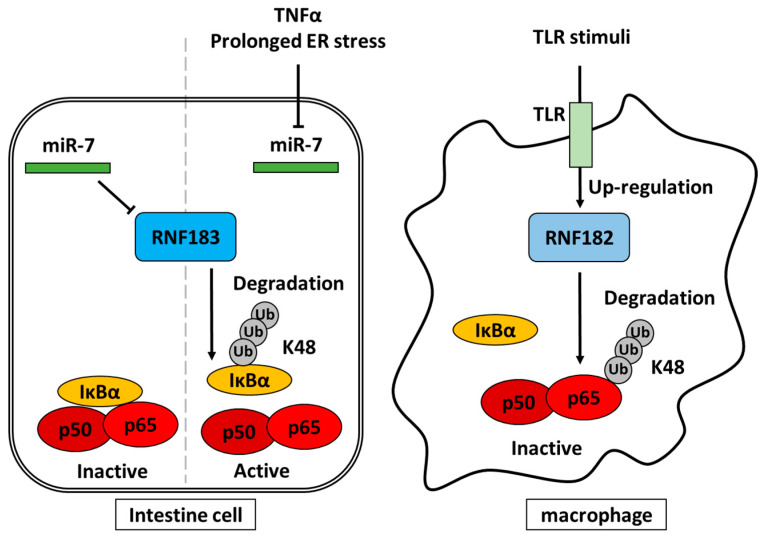
RNF183 and RNF182 are involved in the regulation of the NF-κB pathway. First, RNF183 ubiquitylates IκBα. Then, the ubiquitylated IκBα is degraded, and next, the NF-κB pathway is activated. The miR-7 suppresses the transcription of RNF183. TNFα and prolonged ER stress decrease miR-7, resulting in an increased expression of RNF183. RNF182 ubiquitylates and degrades the subunit p65 of NF-κB, and the TLR stimuli induces the expression of RNF182. Both IκBα and the subunit p65 of NF-κB are ubiquitylated with K48 chain. The arrows indicate activation and the T-bars indicate inhibition.

RNF183 and RNF182 have the opposite effects on the NF-κB pathway in the degradation of IκBα [17] and NF-κB p65 subunit [38], respectively. RNF183 and RNF182 are induced by miR-7 reduction [11,17] and TLR signaling [38], respectively (Figure 3). Therefore, these E3s are not only induced by inflammatory stimuli but also regulate inflammatory responses as a feedback mechanism. Furthermore, RNF183 expression is upregulated by NFAT5 under hypertonic conditions [14]. NFAT5 belongs to the NF-κB/Rel family and is involved in inflammatory responses like NF-κB [58]. Since hyperosmotic response genes include a number of inflammatory genes, which are upregulated by NFAT5, RNF183 may play important roles in inflammation as well as hypertonic stress response. Recently, the relationship between osmotic stress and inflammation was suggested [71]. Thus, RNF183 may be a key factor that links osmotic stress to inflammation. In fact, since it has been reported that osmotic stress exacerbates IBD [72], the osmotic stress response associated with RNF183 may play an important role in the pathology of IBD. Therefore, it is anticipated that substrates of RNF183 related to osmotic stress in IBD will be identified.

## 8. Conclusions

Members of the RNF183 family are widely expressed in the ER, Golgi apparatus, endosomes, and lysosomes. Although their substrates do not overlap with each other, they are involved in some common signaling pathways, such as mTORC1 and NF-κB (Table 1). The tissue expression pattern of these E3s is restricted, but overlaps in some tissues, such as the kidney (RNF183, RNF152, and RNF186), the nervous system (RNF152 and RNF182), and the colon (RNF186 and RNF183) [10]. Thus, it is possible that these E3s could function competitively or cooperatively in the regulation of common pathways. The tissue- and development-specific expression of the RNF183 family suggests that it plays an essential role in specific biological functions. To address the problem of dissociated phenotype in the development of knockout mice [43] and species other than mammals [41,42], it is necessary to establish mice with the knockout of two or more genes. It is also important to investigate whether the RNF183 family works in a coordinated and compensatory manner. On the other hand, it is very difficult to identify the substrates of E3, and experiments involving overexpression alone cannot reveal the true physiological function of a substrate. Therefore, the development of technology for identifying in vivo substrates will be important.

## Figures and Tables

**Table 1:** Features of RNF183 family.

Gene	Cellular Localization	Expressing Tissue	Induction Mechanism	Substrate Protein	Associated Signaling Pathway	Types of Ubiquitin Chain	E2	Associated Disease/Biological Function
*RNF183*	ER, Golgi, lysosome [11,12,13]	kidney, testis [10]; renal medullary collecting duct [13]	prolonged ER stress [11]; NFAT5 [13,14]	BIK [15]; BNIP3L [16]; Bcl-xL [11]; IκBα [17]; DR5 [18]; Na,K-ATPase β1 subunit [19]	apoptosis [11,14,15,16,18]; NF-κB [17,20,21]	K48 [11,15,16,17]; K63 [18,19]	Ubc5c (in vitro) [11]; UbcH5c (in vitro) [12]	IBD [17,18]; endometrial carcinoma [22]; colorectal cancer [15,16,20]; Ewing Sarcoma [21]
*RNF186*	ER [23]; lysosome? [24]	lower gastrointestinal tract, kidney [10]		BNip1 [23]; Occludin [25]; Sestrin-2 [24]	apoptosis [23,26]; mTORC1 [24]	K29 [23]; K48 [24,25]; K63 [23,24]		IBD [25,27,28,29,30,31,32,33]; CKD [34]
*RNF182*	lysosome [35]	nervous system (cortex, hippocampus, cerebellum, spinal cord) [10,35]	oxygen and glucose deprivation [35]; MeCP2 mutation [36]; ischemia-reperfusion injury [37]; TLR stimuli [38]	ATP6V0C [35]; NF-κB p65 subunit [38]	apoptosis [35]; mTORC1 [37]; NF-κB [38]	K48 [35,38]	Ubc5a (in vitro) [35]	AD [35]; Rett syndrome [36]; colorectal cancer [39]; myocardial ischemia [37]
*RNF152*	lysosome [40]	kidney [10]; eyes, neural tube [41]; floor plate [42]	FoxA2 [42]	RagA [43]; Rheb [44]	apoptosis [40]; mTORC1 [42,43,44]; Notch [41]	K48 [40]; K63 [43]; mono [44]	UBC13 (in vivo) [43]; Ubc5a (in vitro) [40]	breast and prostate cancer [45]; colorectal cancer [44,46,47,48]; development of the eyes, midbrain and hindbrain (zebrafish) [41]; proliferation of floor plate cells [42]

## Abbreviations

AD	Alzheimer’s disease
ATP6V0C	V-type proton ATPase 16 kDa proteolipid subunit
Bcl-xL	B-cell lymphoma extra large
BIK	Bcl-2-interacting killer
BNip1	Bcl2/adenovirus E1B 19-kDa interacting protein 1
BNIP3L	cl-2/adenovirus E1B 19 kDa protein-interacting protein 3-like
CD	Crohn’s disease
CKD	chronic kidney disease
CRC	colorectal cancer
DR5	Death receptor 5
DSS	dextran sulfate sodium
DUB	deubiquitinase
E1	ubiquitin-activating enzyme
E2	ubiquitin-conjugating enzyme
E3	ubiquitin ligase
EC	endometrial carcinoma
ER	endoplasmic reticulum
EWSR1-FLI1	Ewing sarcoma breakpoint region 1-Friend Leukemia Integration 1
FATE1	fetal and adult testis-expressed 1
GAP	GTPase-activating protein
GATOR	GAP activity toward RAGs
GCL	ganglion cell layer
GWAS	genome-wide association study
hpf	hours post-fertilization
IBD	inflammatory bowel disease
IκB	Inhibitor of NF-κB
INL	inner nuclear layer
IRE1α	Inositol requiring 1α
IRS1	insulin receptor substrate 1
JNK	c-Jun N-terminal kinase
MeCP2	methyl-CpG-binding protein 2
MHB	midbrain–hindbrain boundary
mIMCD	mouse inner medullary collecting duct
MIRI	myocardial ischemia–reperfusion injury
mTORC1	mammalian target of rapamycin complex 1
NFAT5	Nuclear factor of activated T cells 5
NF-κB	nuclear factor-kappa B
NT2	Ntera2
ONL	outer nuclear layer
Shh	Sonic hedgehog
TLR	Toll-like receptor
TNBS	trinitrobenzene sulfonic acid
TNF-α	Tumor necrosis factor-α
UC	ulcerative colitis
UPR	unfolded protein response
Ub	Ubiquitin
